# Carbon myopia: The urgent need for integrated social, economic and environmental action in the livestock sector

**DOI:** 10.1111/gcb.15816

**Published:** 2021-08-29

**Authors:** Matthew Tom Harrison, Brendan Richard Cullen, Dianne Elizabeth Mayberry, Annette Louise Cowie, Franco Bilotto, Warwick Brabazon Badgery, Ke Liu, Thomas Davison, Karen Michelle Christie, Albert Muleke, Richard John Eckard

**Affiliations:** ^1^ Tasmanian Institute of Agriculture University of Tasmania Burnie TAS Australia; ^2^ Faculty of Veterinary and Agricultural Sciences University of Melbourne Parkville Vic. Australia; ^3^ CSIRO Agriculture and Food St Lucia Qld Australia; ^4^ NSW Department of Primary Industries/University of New England Armidale NSW Australia; ^5^ NSW Department of Primary Industries Agricultural Research Institute Orange NSW Australia; ^6^ Hubei Collaborative Innovation Centre for Grain Industry/School of Agriculture Yangtze University Jingzhou China; ^7^ Livestock Productivity Partnership University of New England Armidale Australia

**Keywords:** adaptation, carbon dioxide removal (CDR), carbon neutral, climate change, emissions intensity, maladaptation, multidisciplinary, policy, socio‐economic, sustainable development goals

## Abstract

Livestock have long been integral to food production systems, often not by choice but by need. While our knowledge of livestock greenhouse gas (GHG) emissions mitigation has evolved, the prevailing focus has been—somewhat myopically—on technology applications associated with mitigation. Here, we (1) examine the global distribution of livestock GHG emissions, (2) explore social, economic and environmental co‐benefits and trade‐offs associated with mitigation interventions and (3) critique approaches for quantifying GHG emissions. This review uncovered many insights. First, while GHG emissions from ruminant livestock are greatest in low‐ and middle‐income countries (LMIC; globally, 66% of emissions are produced by Latin America and the Caribbean, East and southeast Asia and south Asia), the majority of mitigation strategies are designed for developed countries. This serious concern is heightened by the fact that 80% of growth in global meat production over the next decade will occur in LMIC. Second, few studies concurrently assess social, economic and environmental aspects of mitigation. Of the 54 interventions reviewed, only 16 had triple‐bottom line benefit with medium–high mitigation potential. Third, while efforts designed to stimulate the adoption of strategies allowing both emissions reduction (ER) and carbon sequestration (CS) would achieve the greatest net emissions mitigation, CS measures have greater potential mitigation and co‐benefits. The scientific community must shift attention away from the prevailing myopic lens on carbon, towards more holistic, systems‐based, multi‐metric approaches that carefully consider the *raison d'être* for livestock systems. Consequential life cycle assessments and systems‐aligned ‘socio‐economic planetary boundaries’ offer useful starting points that may uncover leverage points and cross‐scale emergent properties. The derivation of harmonized, globally reconciled sustainability metrics requires iterative dialogue between stakeholders at all levels. Greater emphasis on the simultaneous characterization of multiple sustainability dimensions would help avoid situations where progress made in one area causes maladaptive outcomes in other areas.

## Graphical abstract

The majority of global livestock GHG emissions occur in low‐ and middle‐income (LMIC) countries, yet most previous GHG mitigation interventions have been designed for developed countries. There is an urgent need for holistic systems approaches for examining wider environmental, economic and social co‐benefits and trade‐offs associated with GHG emissions mitigation. This is particularly the case for LMIC, given 80% of the growth in demand for livestock products in the coming decades will occur in developing nations. Future GHG mitigation research should aim to examine multiple sustainability metrics, rather than focussing on carbon in isolation.

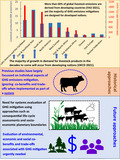

## 1 INTRODUCTION

Contemporary scientific literature is laden with calls to reduce livestock greenhouse gas (GHG) emissions to limit dangerous climate change (Alcock et al., 2015; Harrison et al., 2014; Harrison, Cullen, Tomkins, et al., 2016; Harrison, Jackson, et al., 2014; Ho et al., 2014; Rawnsley et al., 2018; Schellnhuber et al., 2006). *Ceteris paribus* demographic trends suggest that agricultural productivity must increase substantially to meet the demands of a burgeoning and increasingly affluent global population (Struik et al., 2014). These apparent contradictions occur against a background of increasing frequencies of extreme climatic events that impact feed supply, water quality and resources, animal health, infrastructure, profitability and smallholder livelihoods. Together, these factors make the sustainable provision of safe and affordable livestock products increasingly difficult (Bell et al., 2013, 2015; Chang‐Fung‐Martel et al., 2017; Godde et al., 2021; Harrison et al., 2016, 2017).

Policies designed to achieve net zero or net negative emissions to stabilize the global climate by the end of the 21st century (IPCC, 2014; Schleussner et al., 2016) may limit the potential for increased food production as well as the continued support of rural livelihoods in the decades ahead (Frank et al., 2017; Hasegawa et al., 2015; Smith et al., 2013). Large‐scale afforestation and the use of biomass for energy production (Frank et al., 2017; Kreidenweis et al., 2016; Popp et al., 2017) together with population growth will further exacerbate competition for land and food supply.

There are also questions about the applicability of existing GHG emissions mitigation strategies across livestock farming systems (e.g. intensive industrialized livestock production vs. extensive rangeland grazing systems), regions and demographics (FAO, 2019). This is particularly important in low‐ and middle‐income countries (LMIC), where the consumption of animal‐derived products is growing rapidly: between 1970 and 2012, demand for animal products increased fourfold (FAO, 2019). Collectively, these issues elicit polarizing questions of how mitigation of GHG from agricultural systems can take place while productivity is sustainably increased to meet the needs of a growing global population.

The pipeline of sustainable intensification solutions for livestock production systems ranges from theoretical and anecdotal, to proposed, adopted and proven. Sustainable intensification is an intervention that proposes greater agricultural output while keeping the ecological footprint as small as possible (Struik et al., 2014), though multidisciplinary assessments of sustainable intensification strategies are uncommon. In this article, we examine intervention options through a lens of *emissions reduction* (ER) or *carbon sequestration* (CS): the former reduce status quo emissions (e.g. vaccines to reduce enteric methane (CH_4_)), while CS options counterbalance GHG emissions per unit area by withdrawing CO_2_ from the atmosphere (e.g. planting trees on farm to compensate for livestock CH_4_ emissions). ER technologies and practices can only reduce absolute emissions to zero, while CS interventions transfer carbon from the atmosphere into organic forms. Collectively, CS options can exceed the absolute GHG emissions associated with a livestock product, such that net GHG emissions are negative. The combination of ER and CS technologies provides significant potential to maintain or increase livestock productivity while reducing net GHG emissions, which together would reduce emissions intensity (carbon footprint) of livestock products.

While the past work has explored ER and CS practices *en masse*, most studies focus either on knowledge, technologies or mitigation potential in isolation (e.g. the effects of a feed additive on enteric CH_4_ fermentation) using a limited number of metrics (e.g. GHG emissions and livestock production). Few studies explore the co‐benefits and trade‐offs associated with GHG mitigation on environmental, economic and social factors (Mayberry et al., 2019), such as nutrient leaching, soil erosion, biodiversity, profitability, social licence to operate and other sociocultural factors. These sustainability dimensions underpin the United Nations Sustainable Development Goals (SDGs), which aim to end individual priorities and encourage development within a social, economic and environmental nexus (Focus, 2021).

Here, our objectives were to (1) examine the global distribution of livestock production and highlight regions responsible for the greatest livestock GHG emissions, (2) explore co‐benefits and trade‐offs associated with economic, environmental and social implications of GHG emissions mitigation, (3) critique current approaches used to quantify and report livestock GHG emissions and (4) highlight opportunities for future GHG emissions mitigation research.

In the following sections, we first outline global livestock production and projected changes in GHG emissions trends, including differences expected between developed countries and LMIC. We then summarize a range of economic, environmental and social co‐benefits and trade‐offs associated with ER and CS interventions, giving special attention to co‐benefits associated with CS in soils and vegetation. The case for cross‐sector, multi‐metric, multiscale holistic systems‐based emissions mitigation foci is then described, including harmonized solutions for a way forward. We end briefly on research and development (R&D) funding associated with GHG emissions mitigation, including variability in funding cycles.

## 2 WHY ARE EMISSIONS INTENSITIES OF LIVESTOCK PRODUCTS GENERALLY HIGHER THAN THOSE OF OTHER FOODS?

Livestock production systems have been criticized for many reasons in the literature, including their contribution to the loss of terrestrial biodiversity (Steinfeld et al., 2006), their disproportionately low contribution of dietary energy and protein (18% and 39%, respectively) relative to their land‐use footprint (pasture accounts for 68% of agricultural land, plus one‐third of cropland is used for animals (Alexander et al., 2015)), competing use of crop production for feed, impact on nutrient surpluses (Menzi et al., 2010; Stehfest et al., 2013), contribution to deforestation (Smith et al., 2013) and detrimental impacts on ecological resilience (Bouwman et al., 2002). A key focus in recent times has been the contribution of livestock to global GHG emissions and climate change (Rose & Lee, 2008; Schellnhuber et al., 2006).

**FIGURE 1:**
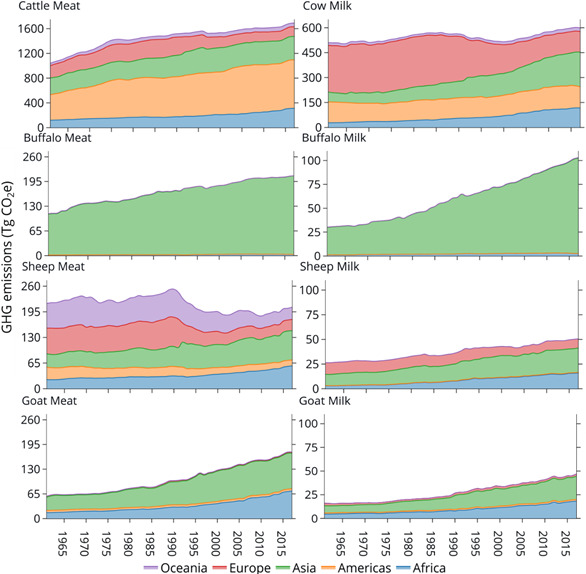
Global GHG emissions from ruminant livestock (cattle, buffalo, sheep and goats). Estimates were computed using IPCC Tier 1 Guidelines associated with on‐farm emissions. Note different scaling on each ordinate axis. Values shown have been adapted from FAOSTAT (http://www.fao.org/faostat)

Globally, livestock are responsible for around 14.5% of total anthropogenic GHG (Gerber et al., 2013) and 70% of total emissions from agriculture, forestry and other land use (AFOLU; Caro et al., 2018). Emissions attributed to livestock systems include carbon dioxide (CO_2_) from land use change and burning of fuels, nitrous oxides (N_2_O) from fertilizers and methane (CH_4_) from manure and enteric fermentation (FAO, 2021a). Beef and dairy cattle are responsible for the majority of emissions, contributing 64–78% of emissions from the livestock sector (Figure 1). The majority of cattle emissions are derived from land use change (45%) and enteric fermentation (39%), with smaller contributions from manure storage, meat processing and transportation.

The emissions intensity of livestock products is typically greater than other foods due to low land use efficiency, production of enteric CH_4_ (if ruminant based, noting that CH_4_ contributes 28 times the global warming of CO_2_ 1) and emissions associated with land conversion for grazing and/or production of animal feed (Ritchie & Roser, 2020). Emissions from ruminants are higher than those from monogastrics because ruminants generally consume lower quality feeds (Herrero, Havlík, et al., 2013) and have lower feed conversion efficiencies (e.g. the quantity of feed required to produce one kilogram of beef or lamb/mutton is around 25 kg or 15 kg, respectively, whereas that for pork and poultry is 6.4 kg and 3.3 kg, respectively, Alexander et al., 2016; Ritchie & Roser, 2020). Although emissions intensities for agricultural products vary widely across commodities, region and production systems, averaged globally and across the supply chain, beef, lamb/mutton, pig meat, poultry meat, dairy milk emissions intensities are 50, 20, 7.6, 5.7 and 3.2 kg CO_2_‐e/kg food product respectively. These contrast with the much lower emissions intensities of plant‐based products including wheat, maize, pulses and root vegetables, which have emissions intensities of less than 0.8 kg CO_2_‐e/kg product (Poore & Nemecek, 2018).

Does this mean that we should cease all livestock production systems? Perhaps not, at least until we have more carefully explored the *raison d'être* of livestock systems, including biophysical, economic, social and cultural reasons, and considering the fact that global variability in emissions intensities of ruminants is greater than most other foods. For example, the production of 100 g of beef protein is responsible around 25 kg CO_2_‐e, but this number hides enormous variability, with 10th and 90th percentiles ranging from 9 kg to 105 kg CO_2_‐e respectively (Poore & Nemecek, 2018). This variability—even among producers in similar geographic regions—implies substantial potential to mitigate environmental impacts and enhance the productivity of the food system (Poore & Nemecek, 2018).

## 3 WHY DO WE NEED LIVESTOCK?

If livestock production systems ‘contribute to environmental problems on a massive scale’ (FAO, 2006a), why are they still practiced today? For many rural communities, livestock satisfy a variety of societal, economic and environmental needs. In addition to the production of food and fibre, livestock provide income, draught power, nutrient recycling through manure and have important social and cultural roles that need to be considered (Steinfeld et al., 2003). For example, livestock have important societal values, such as status or dowry (bridal gifts), and are a vital part of many religious and cultural ceremonies such as Eid al Adha, the Muslim festival of sacrifice. As well, some religions prefer red meat over white meat, and Islamic and Jewish faiths forbid consumption of pork (Brondz, 2018; Larson, 2000; Nakyinsige et al., 2012; Rohman & Che Man, 2012).

Livestock are a fundamental component of the economies of LMIC, providing nourishment and income for rural communities, especially women and nomadic populations (Kristjanson et al., 2014; Rubin et al., 2010). It follows that livestock are a crucial asset and safety net for a large portion of the world. In many regions, livestock comprise the main if not only capital reserve of farming households, serving as a strategic and fluid reserve that reduces risk, provides food and non‐food products (e.g. wool, hides and skin (Thornton, 2010)), and facilitates income diversification and flexibility in response to changing economic and environmental conditions (Kristjanson et al., 2014; Rubin et al., 2010).

In drier regions where aridity is too high for reliable crop production, livestock are often the only viable option (Thornton, 2010; Thornton & Gerber, 2010). Of the 51 million km^2^ of habitable global land, livestock occupy 40 million km^2^ (Mottet et al., 2017; Ritchie, 2019), including grazing land and arable land used for animal feed production (Ritchie & Roser, 2020). Although short‐cycle monogastric production systems such as chickens and pigs can be important for household food security and immediate cash flow, it is worth stressing that only ruminants are able to convert fibrous material and forages—that would otherwise have little or no alternative use—into valuable products (FAO, 2019). Each year, ruminants convert some 2.7 billion metric tonnes of grass dry matter, of which 65% grows on land unsuitable for crops, into edible human protein (Mottet et al., 2017). In developed countries, there is a negative, near‐linear relationship between cost of production and proportion of pasture in the diet (Dillon, 2007), implying that diets comprised by greater proportions of grazed material are conducive to greater profitability.

If a new food production system was proposed to replace or displace livestock on lands unsuited to crops, the new system should also be subjected to environmental and GHG emissions audits, with the emissions intensity of the new product compared with that from the preceding livestock system, while appropriately accounting for the multifunctional values of some livestock systems (Weiler et al., 2014). A fundamental flaw of many past studies has been the lack of comparison of emissions associated with livestock products with alternative (but realistic) agricultural products derived from the same environment. Such assessments can be conducted using *consequential life cycle assessments*, and are the focus of a later section in this article.

As explained below, these essential socio‐economic, environmental and cultural roles of livestock are becoming increasingly important as the sector grows (Herrero, Grace, et al., 2013). The dependence of millions of poor people on livestock suggest that climate change mitigation policies involving livestock must be designed with extreme care (Havlík et al., 2014). Any political incentive intended to reduce livestock supply or demand should first very carefully consider the global distribution of livestock as a function of the biophysical, socio‐economic and cultural services they provide, particularly in locations where livestock underpin the necessities of life.

## 4 HOW (AND WHERE) WILL GLOBAL LIVESTOCK SUPPLY, DEMAND AND GHG EMISSIONS CHANGE IN FUTURE?

**FIGURE 2:**
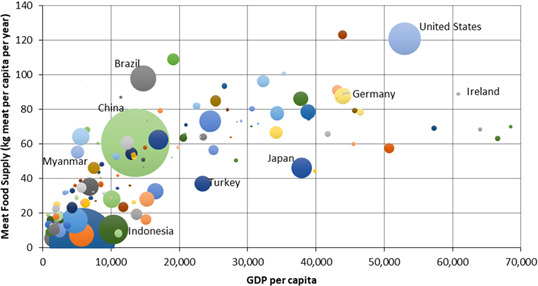
Average meat supply per capita (kilograms per annum) versus gross domestic product ($US GDP) per capita in 2015. Meat supply was computed as the total amount of the commodity available for human consumption during the reference period. Bubble size is proportional to population per country (China = 1,407 million, United States = 321 million and Ireland= 4.7 million). Values shown do not include fish or seafood (adapted from FAOSTAT http://www.fao.org/faostat/en/#data/FBS)

Increasing human population and the global trend towards urbanization will drive increasing consumption of livestock products over the next 20 years (FAO, 2006a, 2006b), particularly in LMIC (Herrero et al., 2016). With rising affluence, the consumption of milk, meat and eggs tends to increase (Figure 2), contributing to improved nutrient status and health of vulnerable populations (Steinfeld et al., 2003). In contrast, increasing incomes in developed countries are no longer associated with increasing animal product consumption. Indeed, higher incomes in developed countries have led to a steady decline in animal food consumption associated with health concerns such as heart and blood circulation diseases (Rae & Nayga, 2010).

**FIGURE 3:**
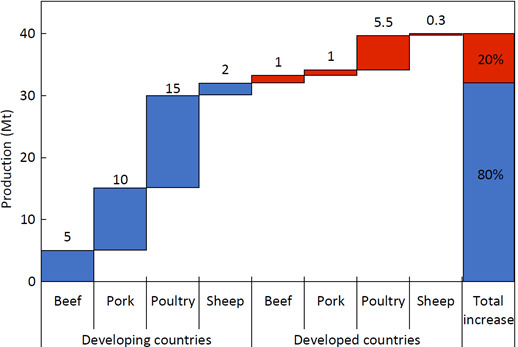
Projected growth in global livestock meat production (carcass weight) in developed countries and LMIC between 2017 and 2029 (adapted from OECD, 2021)

Dietary shifts in the emerging economies of Asia and Latin America have placed unprecedented demand on animal products (Godde et al., 2018). Beef and milk production have more than doubled over the last 40 years, with monogastric production (pigs and poultry) in some areas growing by a factor of five or more (Thornton, 2010). In 2019, global meat production was 340 Mt per annum (>35% poultry meat, 35–40% pig meat and 22% for beef and buffalo meat combined), with production dominated by Asia (144 Mt), followed by Europe (64 Mt) and North and South America (52 and 46 Mt respectively; FAOSTAT, 2020). Meat production is expected to rise to 366 Mt by 2029 (FAOSTAT, 2020). Similarly, the 852 Mt of milk produced globally in 2021 is expected to grow at 1.6% per annum to 997 Mt by 2029, faster than many other agricultural commodities (Figure 3).

**FIGURE 4:**
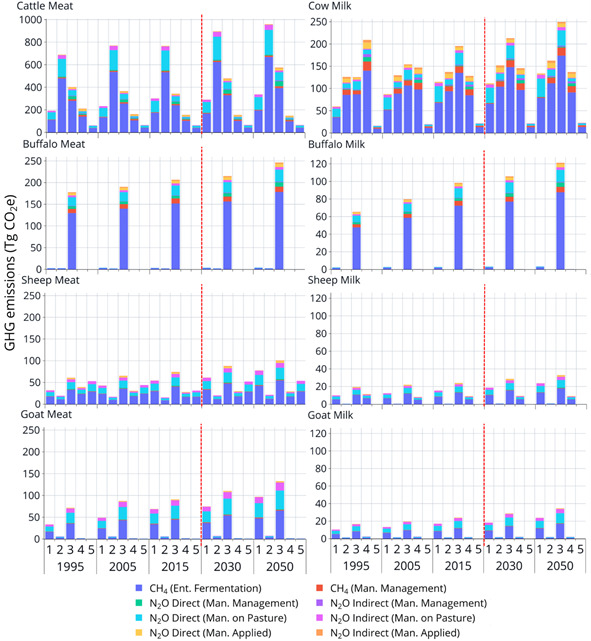
Disaggregated historical and future greenhouse gas emissions (GHG) associated with meat and milk production from key ruminant species (cow, sheep, goat and buffalo) for the main livestock producing continents from 1995 to 2050. *X*‐axis values: 1 = Africa, 2 = Americas, 3 = Asia, 4 = Europe, 5 = Oceania. Values shown to the right of the dashed red line indicate future projected GHG emissions. Note differing scales on ordinate axes. Values shown were estimated using IPCC Tier 1 associated with on farm emissions (adapted from FAOSTAT http://www.fao.org/faostat)

Globally, 66% of livestock emissions are produced by Latin America and the Caribbean, East and southeast Asia and south Asia (FAO, 2021b). Latin America and the Caribbean produce nearly 1.9 Gt CO_2_‐e/annum associated with beef production and land‐use change (deforestation and pasture expansion). East and Southeast Asia are the second largest emitter (1.6 Gt CO_2_‐e/annum), followed by South Asia (1.5 Gt CO_2_‐e/annum). North America, Western Europe, East and North Africa each produce around 0.6 Gt CO_2_‐e/annum, but these African regions produce less than half the protein of North America and Western Europe (FAO, 2021b). Sub‐Saharan Africa produces around 0.4 Gt CO_2_‐e/annum. Globally, emissions from livestock systems increased by 51% between 1960 and 2010 due to growth in livestock production (FAO, 2021b); emissions growth is projected to continue until at least 2050, *ceteris paribus* (Figure 4). The majority of emissions growth has historically occurred in Asia, Africa and the Americas, primarily associated with ruminant production (Figure 1).

**FIGURE 5:**
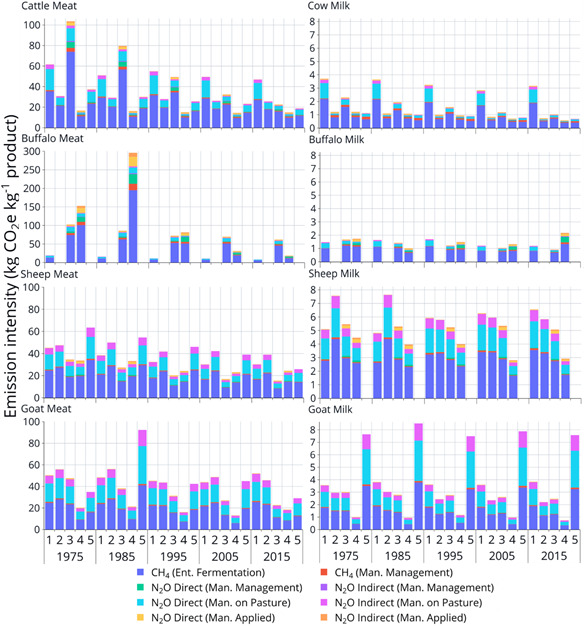
Emission intensities of meat and milk from key ruminants (cattle, buffalo, sheep and goats) for the main livestock producing continents from 1975 to 2015. X‐axis values: 1 = Africa, 2 = Americas, 3 = Asia, 4 = Europe, 5 = Oceania. Values shown were estimated using IPCC Tier 1 associated with on farm emissions. Note differing scales on ordinate axes (adapted from FAOSTAT http://www.fao.org/faostat)

Growth in livestock emissions has been influenced by many interacting socio‐economic, biophysical, political and environmental factors. Asian and African regions have each increased livestock GHG emissions since 1970, but for different reasons. In China, government policies, growing corporate integration and urbanization have encouraged meat and milk consumption (Bai et al., 2018). In contrast, livestock production in the low‐income regions of Africa has been driven by higher animal numbers associated with an expansion of grazing areas with low agroecological potential, coupled with low market access, reduced government investment and high labour costs (Godde et al., 2018). For these reasons, higher livestock production in Africa has been accompanied by higher GHG emission intensities for some livestock products (Figure 5), increasing the emissions from the livestock sector. In contrast, GHG emissions from livestock in developed countries have decreased by 23% from 1960 to 2010 (Caro et al., 2014). Many countries in Europe and Oceania have transitioned towards extensification (similar production per unit area from a greater total area) and deintensification (lower per unit area production) and to ethically and environmentally friendly livestock products, decreasing either gross GHG (Figure 4) and/or emission intensities (Figure 5). Such trends underscore an urgent need for significant improvement in livestock production efficiency in LMIC. This challenge is underpinned by the need to sustainably increase animal production without degrading natural capital or harming the global environment (Box 1).

BOX 1 **One of the greatest challenges of the 21st century**

Satisfying the growing demand for animal products while sustaining the natural resource base (soil, water, air and biodiversity) is one of the foremost challenges facing the world today (FAO, 2019, 2021a). Future global agriculture will be increasingly driven by trends in the livestock sector, many of which are already apparent (FAO, 2006a, 2019, 2021a):

1. Livestock production will become more prone to pests and infectious zootonic diseases, which may have implications for public health such as the COVID‐19 pandemic that devastated global health and economies from 2019;
2. Livestock production systems will shift from local multipurpose activities towards intensive, market‐oriented and increasingly integrated supply chains;
3. Consumption and competition (domestic, national and international) for shared natural resources will increase;
4. Agricultural systems will transition towards large‐scale industrial systems situated near urban centres, but also towards sources of feedstuff, whether crops or trade hubs where feed is imported;
5. Pig and poultry production will increase in importance relative to ruminant production, tending to drive down emissions intensities from the livestock sector; and
6. The majority of growth in meat production in the next 20 years will occur in Asia and the Americas.

Meeting these challenges will require concerted efforts in global and public policy, designed to improve equity, alleviate poverty and improve environmental, public health and food safety. Such efforts must be underpinned by international coordination with strong leadership.

## 5 ECONOMIC, SOCIAL, ENVIRONMENTAL AND BIOPHYSICAL CO‐BENEFITS AND TRADE‐OFFS CAUSED BY GHG EMISSIONS MITIGATION INTERVENTIONS

**FIGURE 6:**
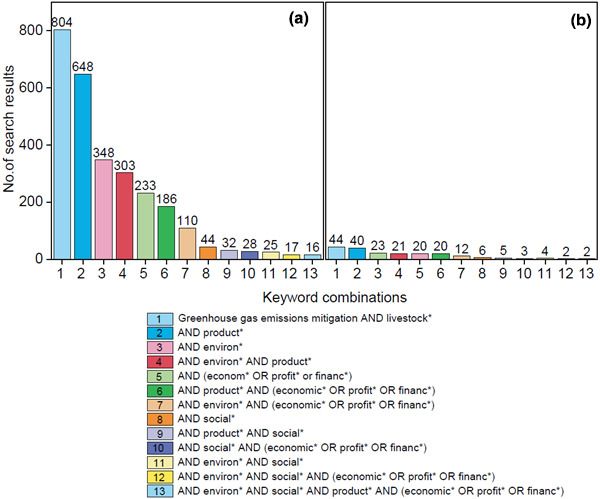
(a) Number of Web of Science Core Collection documents published between 1945 and 2021 that include keywords ‘greenhouse gas emissions mitigation and livestock’ and/or additional biophysical, economic, environmental and social keywords; (b) corresponding search terms for LMIC or developing countries conducted using the search terms ‘AND (LMIC OR (low AND Middle AND income) OR “developing countr*”)’. Bar colours in (b) represent corresponding search colours in (a); note change in order of histogram bars

To examine the extent to which previous work has been conducted with multidisciplinary collaboration, we searched the Web of Science Core Collection database for documents produced between 1945 and 2021, incrementally adding terms to the baseline ‘greenhouse gas emissions mitigation AND livestock’ search. Results are shown in Figure 6a.

Adding the term ‘product*’ reduced the search results from 804 to 648 (where ‘*’ represents a wildcard such that any text containing ‘product’ will obtain a result, e.g. production, productivity etc.). Our search revealed a lack of studies canvassing two or more disciplines associated with livestock GHG emissions mitigation. Many studies focussed on technological aspects, such as mitigation potential, livestock productivity and management of land and/or livestock.

Papers including environmental aspects were lacking (reduction in search results from 804 to 348 in Figure 6a) but perhaps most stark was the dearth of social studies of livestock GHG emissions mitigation, with only 44 relevant documents retrieved. There were even fewer reports of concurrent economic, environmental and social aspects associated with livestock GHG emissions mitigation (Figure 6a).

**FIGURE 7:**
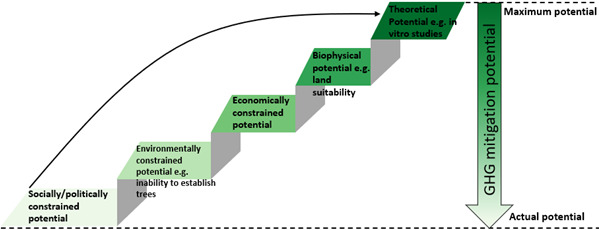
Relationship between theoretical maximum and actual GHG mitigation potential as influenced by social (barriers to adoption, animal welfare, social licence etc.), political (carbon price, red tape, emission mitigation legislation etc.), environmental (nutrient leaching, soil erosion, influence on biodiversity, ecosystems services etc.), economic (cost of implementation and relative return on investment etc.) and biophysical factors (climate, soil, geography, location etc). Social, political and environmental issues historically tend to be investigated last in studies of GHG emissions mitigation, whereas GHG emissions mitigation and productivity potentials are often evaluated first. Text adapted from Smith (2012)

To further examine the extent to which transdisciplinary studies have focused on livestock emissions mitigation options for LMICs, we repeated the search in Figure 6a adding the terms ‘AND (LMIC OR (low AND Middle AND income) OR “developing countr*”)’. Searches including the terms ‘economic’, ‘environmental’ or ‘social’, respectively, revealed only 23, 20 and 6 relevant documents from the entire Web of Science database (Figure 6b), clearly signifying a lack of multidisciplinary livestock mitigation studies for LMIC. The limited number of social studies likely reflects the order in which traditional reductionist research historically occurs, first with GHG emissions mitigation and biophysical/productivity potential, and last with environmental, social and political factors (Figure 7).

Using the search results shown in Figure 6, we next reviewed the economic, environmental and social co‐benefits and trade‐offs associated with ER and CS interventions (Table 1). These were grouped and ranked in ascending order of increasing economic, social and environmental benefit within each of the nine mitigation categories examined. Few interventions had high mitigation potential with concurrent economic, environmental and social benefit. By way of example, feeding nitrate to beef cattle in rangeland environments has been advocated as a pathway by which non‐protein nitrogen could be supplemented to cattle in dry seasons to improve productivity (MLA, 2016, 2020). In Australia, feeding nitrate as a supplement to grazing beef cattle is a legislated CS method under the Commonwealth Emissions Reduction Fund (a policy where farmers and landholders earn carbon credit units and financial return for practices that reduce GHG emissions). While nitrate supplementation has GHG emissions mitigation potential in some environments, it can be unprofitable due to limited effects on productivity (Harrison, Cullen, Tomkins, et al., 2016) and may have animal welfare implications through nitrate poisoning if the feeding regime is inappropriate (Table 1; Callaghan et al., 2014, 2021; MLA, 2016).

**TABLE 1:** Economic, environmental and social co‐benefits and trade‐offs associated with emissions reduction (ER) and/or carbon sequestration (CS) interventions in developed countries and LMICs. Economic co‐benefits and trade‐offs (Econ) consider productivity, profitability and opportunity costs. Environmental co‐benefits (Envirn) were attributed based on air, water and land pollution, land degradation and risk of toxicity. Social aspects (Social) were assessed considering technology availability, social license, capacity for adoption, animal welfare and public perceptions. Yellow shaded rows indicate GHG interventions applicable to LMIC and developed countries, white rows indicate interventions primarily applicable in developed countries. Within the nine categories, interventions are ranked in ascending order of positive economic, environmental and social effects. Legend (base of table): High ≥ 30% mitigation; Med = 10–30% mitigation; Low = ≤ 10% mitigation. Pos: positive; Neg: negative; NC: no change; ‘?’ = unclear. Single, double and triple dot points represent low, mid and high mitigation potential, respectively. Green and red dots represent positive and negative changes, respectively

Intervention	Co‐benefits and trade‐offs					Reference
	Type	Mitig	Econ	Envirn	Social	
**1. Animal management or genetics**						
Transient confinement feeding of grazing animals to preserve ground cover and increase liveweight gain	ER, CS	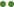	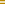	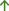	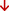	Cottle et al. (2016); Modernel et al. (2013); Molossi et al. (2020)
Genetic selection (residual feed intake) for low CH_4_ production	ER	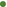	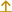	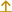	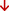	Alcock et al. (2015); Beauchemin et al. (2020); Bezerra et al. (2013); Hristov et al. (2013); Leahy et al. (2019)
Genetic selection for larger adult body size	ER	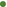	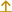	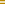	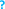	Cottle et al. (2016)
Genetic selection for greater fleece weight production per animal	ER	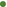	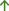	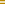	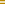	Alcock et al. (2015); Cottle et al. (2016)
Reducing age of first mating	ER	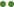	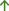	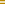	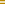	Alcock et al. (2015); Christie et al. (2016); Cullen et al. (2016); Harrison, Christie, et al. (2014)
Extended seasonal lactation duration in dairy cows	ER	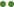	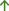	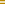	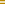	Browne et al. (2014)
Recombination bovine somatotropin to increase growth rates	ER	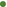	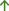	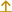	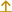	Capper et al. (2008); Capper and Cady (2012); Hristov et al. (2013); MacLeod and Moran (2017)
Optimizing herd structure for improved profit	ER	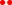	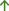	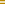	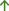	Harrison, Cullen, Tomkins, et al. (2016)
Milking dairy cows less frequently	ER	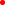	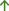	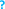	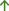	Christie et al. (2014)
Improved animal health	ER	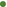	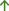	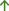	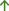	Herrero, Havlík, et al. (2013); Herrero et al. (2015); Shields and Orme‐Evans (2015)
Reduced adult and juvenile mortality at birth	ER	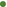	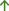	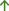	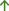	Herrero et al. (2015); Hristov et al. (2013); Shields and Orme‐Evans (2015)
Reduced age at slaughter and days on feed	ER	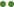	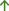	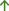	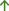	Herrero et al. (2015); Hristov et al. (2013); Shields and Orme‐Evans (2015)
Higher fecundity/higher weaning rates	ER	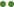	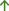	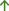	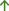	Cullen et al. (2016); Harrison, Christie, et al. (2014); Harrison, Jackson, et al. (2014); Ho et al. (2014)
Increased productivity/ growth rates of young livestock	ER	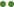	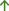	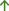	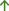	Beauchemin et al. (2020); Harrison et al. (2011a), Harrison et al. (2011b); Harrison et al. (2012a), Harrison et al. (2012b); Hristov et al. (2013); Leahy et al. (2019); Reisinger and Clark (2018); Shields and Orme‐Evans (2015); Taylor and Eckard (2016); Taylor et al. (2016)
**2. Feed additives and feeding management**						
Nitrate feeding in rangeland environments	ER	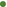	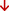	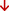	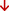	Beauchemin et al. (2020); Cottle et al. (2016); Herrero et al. (2015)
Grape marc	ER	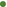	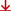	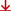	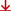	Cortés et al. (2020); Davison et al. (2020)
Nitrification inhibitors	ER	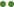	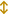	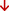	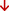	Herrero et al. (2015); Hristov et al. (2013); Kösler et al. (2019); Leahy et al. (2019)
Tannins	ER	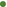	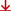	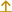	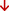	Beauchemin et al. (2020); Herrero et al. (2015)
Chemical inhibitors (3‐nitrooxypropanol)	ER	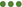	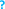	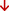	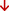	Beauchemin et al. (2020); Herrero et al. (2015); Kösler et al. (2019); Leahy et al. (2019)
Concentrates, e.g. grains (highly digestible feeds)	ER	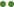	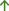	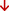	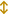	Beauchemin et al. (2020); Herrero et al. (2015); Hristov et al. (2013)
Dietary lipids	ER	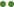	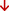	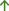	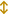	Beauchemin et al. (2020); Herrero et al. (2015); Hristov et al. (2013); Ludemann et al. (2016)
Rumen microbiome and fermentation manipulation	ER	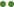	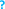	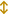	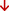	Beauchemin et al. (2020); Hristov et al. (2013); Kumari et al. (2020); Leahy et al. (2019)
Methane vaccine	ER	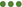	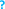	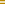	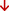	Rolfe (2001)
Algal‐derived	ER	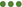	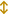	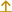	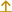	Beauchemin et al. (2020); Davison et al. (2020); Kinley et al. (2020); Roque et al. (2019)
Feedstuffs with low N concentration	ER	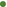	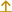	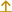	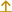	Christie et al. (2014); Leahy et al. (2020); Reisinger and Clark (2018)
Biochar as animal feed supplement	ER	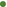	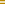	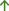	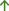	Roberts et al. (2010); Schmidt et al. (2019)
**3. Pasture types and management**						
Birdsfoot trefoil (*Lotus* spp.)	ER, CS	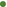	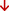	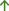	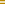	Doran‐Browne et al. (2015)
Biserrula	ER, CS	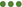	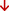	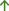	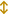	Davison et al. (2020)
Simplified/fewer intensive systems/lower fertilizer use	ER	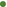	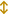	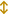	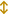	Harrison et al. (2017); Leahy et al. (2019); Reisinger and Clark (2018)
Silages	ER, CS	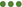	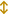	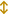	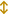	Kumari et al. (2020)
Pasture production improvement	ER	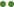	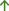	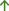	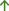	Alcock and Hegarty (2006); Harrison, Christie, et al. (2014); Herrero et al. (2016); Hristov et al. (2013); Kumari et al. (2020); Smith et al. (2007)
Fodder Beet	ER, CS	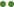	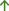	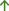	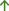	Leahy et al. (2019); Ruis and Blanco‐Canqui (2017); Sun et al. (2016)
Plantain	ER, CS	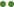	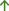	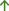	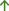	de Klein et al. (2020); Leahy et al. (2019); Luo et al. (2018)
Leucaena	ER, CS	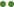	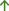	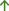	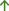	Harrison et al. (2015); Taylor and Eckard (2016); Taylor et al. (2016); Tomkins et al. (2019)
Desmanthus	ER, CS	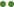	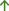	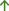	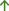	Davison et al. (2020)
Fodder Rape	ER, CS	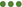	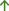	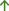	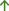	Leahy et al. (2019); Ruis and Blanco‐Canqui (2017); Sun et al. (2016)
**4. Soil management and health**						
Addition of phosphorus fertilizers	ER, CS	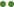	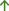	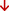	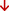	Chan et al. (2010); Cottle et al. (2016); Harrison, Christie, et al. (2014); Leip et al. (2015)
Biochar to improve soil C	CS	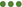	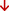	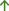	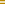	Joseph et al. (2015)
Converting annual crops to permanent pastures	CS	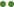	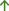	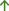	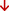	Meier et al. (2020); Meyer et al. (2016); Meyer et al. (2015)
Improving soil carbon under trees	CS	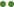	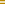	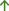	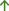	Doran‐Browne et al. (2016)
**5. Manure management**						
Solid liquid separation	ER	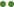	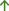	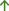	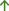	Grossi et al. (2019); Hristov et al. (2013)
Anaerobic digestion	ER	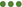	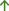	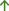	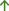	Gerber and Span (2008); Grossi et al. (2019); Hristov et al. (2013)
Decreased storage time	ER	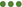	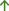	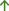	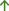	Grossi et al. (2019); Hristov et al. (2013)
Frequent manure removal	ER	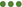	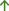	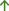	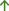	Grossi et al. (2019); Hristov et al. (2013); Thornton (2010)
. **Agroforestry**						
Planting trees on farm	CS	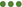	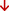	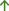	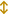	Doran‐Browne et al. (2016); Leahy et al. (2019); Reisinger and Clark (2018)
Forest conservation/Avoided deforestation	CS	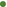	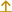	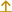	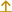	Petersen et al. (2013); Reisinger and Clark (2018); Rivera‐Ferre et al. (2016)
**7. Whole farm and regional management**						
Integrated farming systems	ER, CS	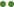	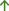	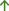	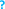	Thornton et al. (2018)
Land restoration/Avoided land degradation	ER	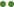	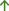	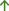	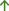	Doran‐Browne et al. (2016); Herrick et al. (2019); Rivera‐Ferre et al. (2016)
**8. Technology and information services**						
Renewable and alternate energy sources	CS	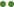	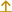	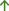	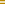	Fleming et al. (2019); Mayer et al. (2020)
More accurate long‐range and seasonal climate forecasts	ER	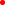	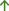	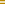	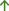	Chang‐Fung‐Martel et al. (2017); Harrison, Cullen, et al. (2017); Thornton et al. (2018)
Digital services and decision support^a^	ER	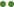	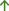	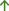	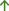	Fleming et al. (2019); Lovarelli et al. (2020); Tullo et al. (2019)
**9. Social aspects**						
Traditional/indigenous knowledge	ER, CS	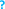	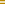	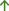	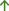	Fleming et al. (2019); Kemp et al. (2013); Thornton et al. (2018)
Cooperativism (Farmer producer organizations)	ER, CS	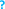	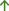	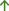	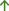	Thornton et al. (2018)

^a^ Satellite imagery, big data, GPS, automation, assessment and decision support.

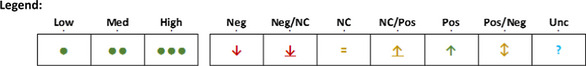

Of the 48 ER interventions shown in Table 1, 18 had triple bottom‐line benefit. All manure management interventions had economic, environmental and social benefit, with most being applicable in LMIC and having high GHG mitigation potential due to their ability to limit both CH_4_ and N_2_O emissions (through effects on methanogenesis and/or denitrification). However, manure management approaches are generally applicable only to intensive production systems such as dairies, feedlots and piggeries in developed countries, and smallholder confined feeding operations and subsistence farming operations in LMIC, because manure must be accessible and manageable. For the majority of grassland‐based systems, it is not feasible to collect livestock faeces and urine because excreta is spread too sparsely. Such areas include the dryland tropics and continental locales of Asia and North America (Petersen et al., 2013; Steinfeld et al., 2006) as well as rangelands in central Australia, the African savannas, the South American Pampas and the Great Plains of North America.

For pasture and animal management, only those interventions designed to increase the growth rates of young livestock (or production efficiency), reduce age at slaughter or increase fecundity/weaning rates had medium–high mitigation potential. Reducing time to slaughter and/or improved growth rates of young animals benefits farmers (1) economically through greater liveweight production, (2) environmentally through lower residence time of animals on farm which, at broader scales, causes less soil erosion and destruction of natural habitats and (3) socially through improved animal welfare and lower mortality rates of juvenile and adult animals. However, any intervention that improves growth rates of young animals only has mitigation benefit if overall stocking rates are not subsequently increased, for example, if animals removed from a farm are not replaced by others. This highlights an important distinction between reductionist mitigation research versus approaches that evaluate livestock emissions as part of a holistic system; only integrated systems approaches capture important trade‐offs between mitigation at the animal and farm level, and between the farm and global level. Such trade‐offs may include efficiency gains at the animal level that are offset by increased numbers of animals on farm over a set duration. ‘Pollution swapping’ is another trade‐off that can be captured by systems approaches, for example, increased N fertilizer can improve pasture growth and SOC sequestration, but this may result in greater N_2_O emissions. Another trade‐off is ‘leakage’, such that emissions mitigation on one farm is offset by increased emissions on another farm (noting that leakage could be temporal, sectoral or geographical). Leakage may include (1) destocking a farm to reduce emissions, leading to increased stocking rates elsewhere to maintain supply and (2) revegetation or bioenergy cropping on pasture land that leads to deforestation elsewhere to provide pasture. Underpinning many of the animal management options that have medium–high mitigation potential is reduced stocking rate. This is particularly important in LMIC and extensive areas, because reduced animal densities facilitate higher feed per animal, improved growth rates, higher soil CS (Zhang, Huang, et al., 2015) and potentially landscape restoration (discussed further below).

Of the 19 CS interventions in Table 1, six had triple bottom‐line benefit, five of which had medium–high potential to reduce emissions. Perennial legumes (e.g. leucaeana and Desmanthus) offer multiple avenues for emissions mitigation for the environments in which they are suited. These include increased soil carbon storage at depth, higher crude protein and mitigation of enteric CH_4_ through condensed tannins (Harrison et al., 2015; Radrizzani et al., 2011; Ruis & Blanco‐Canqui, 2017; Sándor et al., 2020; Suybeng et al., 2019; Tomkins et al., 2019), though in some cases, legumes may increase N_2_O emissions as a result of higher deposition of urinary nitrogen (Harrison, Cullen, Tomkins, et al., 2016; Harrison et al., 2015). Forage brassicas (e.g. forage rape) also have medium–high mitigation potential due to higher digestibility that can lead to reduced enteric CH_4_ per unit dry matter consumed, higher livestock growth rates and potentially increased soil CS (Ruis & Blanco‐Canqui, 2017; Sun et al., 2016; Thomson et al., 2016). But dietary composition is also important: If perennial legumes or forage brassicas form only a small part of annual intake, mitigation of enteric CH_4_ is likely to be small. This observation highlights the important trade‐off between component‐level research (e.g. evaluation of CH_4_ mitigation per unit intake of the forage per se) versus systems approaches that account for both mitigation per unit intake but also other features that influence GHG mitigation, including pasture botanical composition and animal dietary constituents.

The trade‐offs associated with many of the interventions in Table 1 underpin *a clear need for transdisciplinary efforts* in future assessments of mitigation interventions. Many of the interventions have been evaluated only in a theoretical sense and focus entirely on GHG emissions mitigation, thus ignoring important cross‐disciplinary factors such as cost, practicality, reversibility, market access, political factors or social license to operate, which collectively and ultimately define adoptability and impact. Such factors limit the feasible mitigation to a value that is much lower (Figure 7).

The majority of interventions in Table 1 were tested in and *designed for developed countries*. There are fewer mitigation options that have been tested in LMIC. Mitigation options that could be applied in LMIC that have triple bottom‐line benefit include improved animal health and animal husbandry leading to greater growth rates and/or younger age at slaughter, feeding of concentrates, planting trees or perennial legumes (e.g. leucaena) and manure management techniques. Only seven of these interventions have medium or high GHG emissions mitigation potential. Similar to developed countries, not all of these options will be applicable in LMIC due to farming system practices, location, workforce availability, access to markets and agronomic knowledge, cultural aspects and other factors. Cohen et al. (2021) indicate that a greater understanding of the co‐impacts (co‐benefits and adverse side effects) is needed to realize the potential for sustainably meeting multiple objectives, increasing the efficiency and cost‐effectiveness of climate actions (both mitigation and adaptation). They note that the impact of such information is determined by the manner in which it is framed and communicated, and that framing needs to be context‐ and application‐specific.

The majority of GHG mitigation studies with an economic focus were associated with profitability/cost of a given intervention (e.g. research and development costs, capital investment, costs of feeding, effects of carbon price (Cohn et al., 2014; Wall et al., 2010)) or the opportunity costs of using land and other resources to raise animals considering their alternative use for other food or non‐food sources (Garnett, 2009; Wall et al., 2010). We found that more economic studies examine co‐benefits or positive effects associated with GHG mitigation, compared with environmental and social studies (Table 2).

**TABLE 2:**
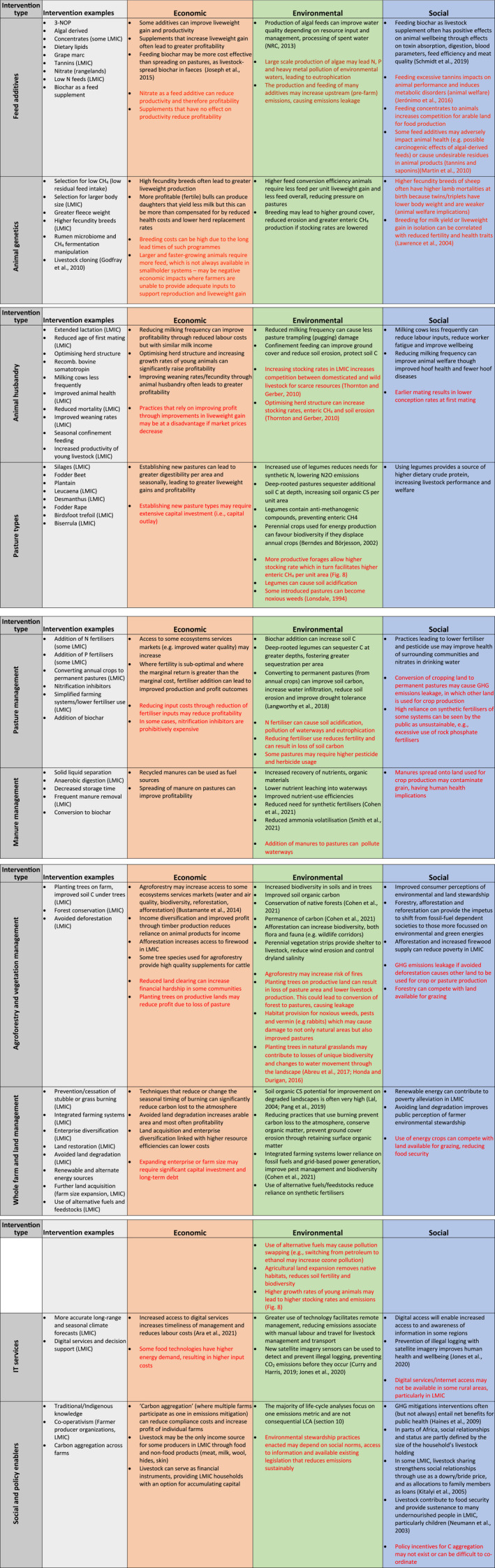
Examples of economic, environmental and social co‐benefits (black) and trade‐offs (red) associated with farm GHG emissions mitigation in the livestock sector in developed countries and LMIC. Interventions that apply in both developed countries and LMIC are shown in parenthesis in the first column; all other interventions apply to developed countries

In contrast to economic studies, environmental studies were broader in scope and tended to be equally focused on the co‐benefits and trade‐offs associated with GHG emissions mitigations (black and red text respectively in Table 2). Assessments ranged from environmental implications caused by the production of algae for livestock feeds, to the effects of trees or deep rooted pastures on soil carbon, biodiversity and natural capital, to planting legumes to replace synthetic nitrogen fertilizers. Studies of manure management were also common but less frequently were focused towards LMIC, with the notable exception of the review by Petersen et al. (2013) who discussed a range of solid and liquid manure management options in Sub‐Saharan Africa, Southeast Asia, China and Europe. The mitigation potential associated with reduced food loss and waste was high (Bellarby et al., 2013). As there are many livestock production and environmental co‐benefits associated with managing soils and vegetation (trees and shrubs), these interventions are given special consideration in the following sections.

As discussed above, there was a *paucity of social studies* compared with other disciplinary areas (Table 2; Figure 6a). Most social studies were related to animal welfare, including health benefits of feed additives, mortality associated with higher fecundity breeds, maladaptive breeding traits and co‐benefits derived from shade and shelter belts. While many studies focused on health or dietary implications associated with GHG mitigation through reduced demand or consumption (e.g. Bajželj et al., 2014; Popp et al., 2010; Tilman & Clark, 2014; Westhoek et al., 2014), few studies focused on participation in carbon markets or motivations/barriers to adoption of mitigation interventions (e.g. Kragt et al., 2017; Torabi et al., 2016). The majority of studies centred on social aspects of carbon farming were conducted in and for developed countries.

Despite considerable variability in the pathways with which co‐benefits and trade‐offs could be realized with individual mitigation interventions, many share common principles:

1. **Economic co‐benefits:**
  - Improved liveweight gain and/or animal productivity: for example, feeding of supplements, higher digestibility feed types, improved pasture species, higher fecundity breeds, productivity co‐benefits associated with shade or provision of shelter;
  - Reduced management/input costs: for example, reducing dairy cow milking frequencies or using less synthetic fertilizers as a result of planting legumes;
  - Income diversification: through access to new markets such as carbon trading, ecosystems services or payments for biodiversity, renewable energy, improvements to water and/or air quality, or the ability to create a new product (e.g. bioenergy crops) or branding (‘carbon neutral’).

- **Economic trade‐offs:**
  - Cost of implementation: for example, the limited effect of some supplements on liveweight gain (e.g. nitrates) or the small effects of nitrification inhibitors on pasture growth do not compensate for the cost of the intervention (Callaghan et al., 2021);
  - Market variability: Reduced prices for animal products, carbon or ecosystems services may result in lower income compared with no intervention;
  - Loss of productive arable land due to, for example, planting trees or restoration of natural habitats;
- **Environmental co‐benefits:**
  - Pasture sustainability: for example, improved ground cover, pasture persistence and production resulting from management practices that facilitate spelling or reduced grazing duration of grasslands (such as seasonal confinement feeding or introducing animal genotypes with greater feed‐conversion efficiency), reduced need of synthetic fertilizers by planting legumes, converting to permanent pastures as opposed to annual crops;
  - Ecosystems services: for example, improved water quality from large‐scale algae production, improved soil carbon from planting deeper rooted pasture species or spreading biochar, reduced leaching of nutrients into waterways through manure management;
  - Reduced need for fossil fuels: preventing/displacing off‐farm GHG emissions, for example, planting of bioenergy crops (Goldemberg, 2007), use of renewable energy on farm such as solar panels (photovoltaics), wind turbines or anaerobic digestors for manure (Bardi et al., 2013; Hernandez et al., 2015).
- **Environmental trade‐offs:**
  - Emissions leakage in which emissions no longer occurring in a location occur elsewhere, for example, emissions caused by the production of feed additives or grain used for animal feeds;
  - Reduced water quality such as leaching of P and N into waterways, causing eutrophication;
  - Reduced soil quality, e.g., acidification caused by excessive use of nitrogenous fertilizers or inclusion of legumes in pasture;
  - New pasture types may become weeds if unmanaged or allowed to escape;
  - Habitat for pests may be engendered if vegetated areas are created (or land spared), providing habitat for vermin, weeds and pests that together may damage both natural and arable areas.
- **Social co‐benefits:**
  - Animal health and welfare through provision of shade and shelter, reduced heat stress, reduced need for labour, improved animal traction in LMIC, improved milking frequencies of dairy cows, improved nutrition (some pasture types and legumes);
  - Human health and welfare associated with reduced exposure to heavy metals (manure management), improved water quality from reduced ground water contamination, increased firewood supply associated with afforestation in LMIC, social relationships in LMIC and improved health of rural communities with the addition of animal protein to human diets;
  - Enhanced resilience of rural communities through additional expenditure on infrastructure such as fencing to manage over‐grazing by livestock and feral herbivores, carbon trading, which provides a consistent source of income during drought, enabling early destocking to minimize the risk of land degradation and hasten pasture recovery post‐drought (Cowie et al., 2019).
- **Social trade‐offs:**
  - Animal health and welfare associated with some feed additives (e.g. potentially carcinogenic effects of algal feeds (Abbott et al., 2020) and tannins (Jerónimo et al., 2016) increased mortalities associated with higher fecundity breeds, maladaptive breeding traits);
  - Adverse societal impacts: caused by fuel/livestock feed competition, competition for land; vegetation management at the expense of food security in the absence of adequate governance; livelihoods impacted where livestock are the main source of income, especially in some LMIC, and pollution swapping (e.g. where livestock GHG emissions mitigation facilitates greater use of fossil fuels elsewhere (Smith et al., 2007)).

Carbon sequestration opportunities provide a pathway for greater emissions mitigation than the potential associated with ER. As detailed further below, two promising emissions CS pathways include CS in soils and in vegetation: These options provide medium–high mitigation potential (Table 1) with ability to offset enteric CH_4_ (Doran‐Browne et al., 2016; Henderson et al., 2015; Leahy et al., 2019; Smith et al., 2020) are widely applicable in developed countries and LMIC, and offer multiple co‐benefits.

## 6 MITIGATION POTENTIAL AND CO‐BENEFITS ASSOCIATED WITH CARBON SEQUESTRATION IN SOILS

Grazing lands (savannas, grasslands, prairies, steppe and shrublands) cover 45% of the earth’s land surface (excluding Antarctica; Ritchie, 2019), such that modest increases in soil organic carbon (SOC) could offset GHG emissions from livestock in grazing lands (Derner & Schuman, 2007; Zhang, Gao, et al., 2015). Recent work has suggested that soil carbon represents 25% of the potential of natural climate solutions, of which 40% is the protection of existing soil carbon, and 60% is rebuilding of depleted stocks (Bossio et al., 2020). Co‐benefits associated with increased soil C in grazing systems include improved plant available water holding capacity and increased nitrogen supply through mineralization (Wander & Nissen, 2004), which may buffer the impact of climate variability and change.

There are several grazing and land management options that can increase SOC (Tables 1 and 2). Grazing practices that improve SOC include those that alter pasture species composition, forage consumption and ground cover, leading to greater nutrient, organic matter or water ingress into pastures (Conant & Paustian, 2002; Lal, 2004). Land management practices that can increase SOC include (a) moving from annual cropping to perennial pastures (Badgery et al., 2014), (b) increasing soil fertility, (c) improving grazing (in some cases reducing stocking rate) and (d) planting trees or deep‐rooted perennials. Altering stocking rate to maximize forage production could result in a global sequestration of up to 148 Tg CO_2_/annum in grazing lands (Henderson et al., 2015; Herrero et al., 2016), while sowing legumes could sequester an additional 203 Tg CO_2_/annum (despite being applicable over a much smaller area; Henderson et al., 2015). However, increased N_2_O emissions from legumes can offset SOC sequestration benefits (Henderson et al., 2015), again suggesting a need to holistically quantify changes in all of the main GHG emissions (CO_2_, N_2_O, CH_4_) whenever an intervention is made.

Because there are many interactions that govern soil carbon flux, grazing often has variable and inconsistent effects on SOC. Grazing perturbs above‐ and below‐ground net primary production, shifting grassland composition (Derner et al., 2006), altering nutrient cycling, groundcover, soil temperature, moisture and respiration. Shifting from heavy to light grazing utilization may increase SOC (Byrnes et al., 2018), with SOC under light grazing often being higher than that with no grazing (Jiang et al., 2020; Liu et al., 2012; Orgill et al., 2012).

More than 80% of the potential increase in SOC is in LMIC, where pasture production would be expected to increase following periodic de‐stocking, resulting in a lower annual average stocking rate (Henderson et al., 2015). East and South Africa in particular have large potential for increased SOC sequestration (0.1–3.1 Mg/ha.annum) and there are reports of up to 90% decline in SOC in the heavily grazed highlands of South Africa (Dlamini et al., 2014). Despite this, very little work has been conducted on the potential of practices or incentives to encourage SOC sequestration in East and South Africa (Tessema et al., 2020).

It is important to note that there are limits to the maximum SOC stocks in any soil. If SOC is already close to its maximum, further increases to SOC will be unlikely. In these cases, effort would be better spent in preventing decline of SOC (Badgery et al., 2020; Sanderson et al., 2020) through the implementation of the approaches outlined above. Lack of continuing nutrient input (e.g. fertilization), drought exposure or other changes relative to historical land management may also enhance the rate of organic carbon conversion to CO_2_ (either through leaching or respiration), causing soil carbon to decline.

There are substantial opportunities to increase SOC on degraded grazing lands, because the most degraded soils often have the highest capacity to store additional SOC. This is most evident in LMIC such as East Africa and southwest China where stocking rates are often excessive, impacting on production and causing degradation (Lohbeck et al., 2018; Michalk et al., 2019; Pang et al., 2019). Of the total global potential in SOC sequestration in grazing lands, the largest potential is in Central/South America (26.7 Tg CO_2_/annum), Sub‐Saharan Africa (24.3 Tg CO_2_/annum), Oceania (15.6 Tg CO_2_/annum) and East/Southeast Asia (13.7 Tg CO_2_/annum; Henderson et al., 2015). While there is moderate variation in regional sequestration rates (0.13–0.32 Mg CO_2_/ha.annum, coefficient of variation = 26%), most of the difference in total carbon storage potential between continents was due to variations in land area (coefficient of variation = 72%). Henderson et al. (2015) also found a relationship between agroecological zone (temperate, humid and arid) and sequestration potential. At the global average level, humid rangelands have the highest C sequestration rates per hectare, followed by arid and temperate rangelands. However, sequestration rates differ to total sequestration potential. Arid areas account for just over half of the total global soil C sequestration potential due to their dominant share of the total rangeland area. At the global level, the largest soil C sequestration potentials lie in Central and South America, Sub‐Saharan Africa and Oceania due to the large areas of rangelands in the humid and arid agroecological zones within these continents. Similarly, the low per hectare potentials in Central Asia, Eastern Europe and Russia, East and Southeast Asia reflect the higher proportions of temperate rangelands in these regions (Henderson et al., 2015).

## 7 MITIGATION POTENTIAL AND CO‐BENEFITS ASSOCIATED WITH CARBON SEQUESTRATION IN VEGETATION

Similar to the CS opportunities afforded by soil carbon, the global CS potential in vegetation is large (Domke et al., 2020). The CS potential associated with reforestation (planting trees where there once was forest) and afforestation (planting trees on previously unforested land) is, respectively, around 1.5–10.1 Gt CO_2_‐e/annum (Smith et al., 2020) and 1.5 Gt CO_2_‐e/annum (Nilsson & Schopfhauser, 1995). The majority of this global carbon sink potential is in grasslands (Scurlock & Hall, 1998).

Forestry (trees only), agroforestry (integrated agriculture and trees) and silvopastoral systems (integrated livestock grazing and trees) are recognized carbon offset activities in some carbon marketing schemes, creating opportunities for landholders in LMIC to generate income through CS associated with vegetation management. Doran‐Browne et al. (2018) showed that planting trees on 20% on a high‐density livestock farm in Australia achieved carbon neutrality. However, after 30 years, CS in the trees had slowed considerably. This result highlights the contrasting transient nature of CS relative to the continuing emissions of livestock (assuming stocking rates remain unchanged), suggesting a need for interventions that are not limited by saturation once carbon stored in soils and vegetation nears its potential. The sequestration ceiling could be avoided by harvesting forests for use in long‐term products, harvesting biomass for bioenergy and producing and applying biochar to soils.

Although agroforestry practices in LMIC are more widespread than in developed countries (Ramachandran Nair et al., 2010), carbon market opportunities in LMIC associated with agroforestry are lower, hampered by insecure land tenure and high transaction costs that are prohibitive for smallholders in Africa and Indonesia, for example (Cacho et al., 2005; Jindal et al., 2008). Widespread participation in carbon markets in the foreseeable future is unlikely without accompanying the development of policy instruments to reward the rural poor for other environmental services such as watershed protection and biodiversity enhancement (Garrity, 2004; Lee et al., 2016). This type of financial reward may help recoup landholder costs incurred in protecting and enhancing environmental services.

Agroforestry provides a range of ecosystems services. Carbon sequestration in soils and vegetation could perhaps be more appropriately viewed as a co‐benefit from improving livestock productivity and ecosystems services, rather than a primary objective for either managing land ecosystems (Henderson et al., 2015; Herrero et al., 2016) or for deriving income from CS *per se* (Alcock et al., 2015; Harrison, Cullen, Tomkins, et al., 2016; Ho et al., 2014). Planting trees can improve livestock production through shade and shelter that regulates the microclimate (Deniz et al., 2019) which potentially improves liveweight gain and survival (Gregory, 1995; Montagnini et al., 2013), while fodder trees and shrubs can be a valuable source of feed in dry seasons (Franzel et al., 2014; Vandermeulen et al., 2018). In dry environments, hydraulic redistribution (Bayala & Prieto, 2020; Bogie et al., 2018) could contribute towards enhanced growth of pasture under some tree species.

In the low productivity rangelands of developed countries, income from “carbon farming” (e.g. avoided deforestation, afforestation and forest regeneration) can enhance the resilience of farming systems and rural communities, facilitating expenditure on infrastructure such as fencing to manage overgrazing by livestock and feral herbivores, providing income during periods of drought, enabling early destocking to minimize risk of land degradation and hasten pasture recovery post‐drought (Cockfield et al., 2019; Cowie et al., 2019). While commercial forestry provides a significant avenue for CS, establishment of industrial plantations in LMIC can displace transhumant pastoralism (Ramprasad et al., 2020) and exacerbating poverty in local communities (Andersson et al., 2016). Commercial forestry companies typically manage multiple stands of different ages to provide a constant supply of products and income. Sawlog rotations are around 25–80 years, pulp rotations 10–15 years and coppiced biomass rotations are 3–5 years, depending on climate. As income is received after harvest, delayed returns present a significant cash flow issue for smallholders who own a limited number of stands. However, landholders who plant woodlots usually do so as investments to supplement income from agricultural enterprises. Indeed, Nigussie et al. (2021) found that integration of short rotation acacia plantations in diversified farming enterprises enhanced livelihoods of some (but not all) smallholders in Ethiopia.

The review comment is applicable to LMIC where international forestry companies develop plantations on leased community land or public land, displacing informal grazing.

Silvopastoral systems provide a range of environmental co‐benefits and livestock co‐production benefits. Leguminous trees in silvopastoral systems can enhance nutrient cycling and improve soil fertility (Lira Junior et al., 2020; Martínez et al., 2014) and globally have significant potential to sequester carbon (see previous section). Moderate tree cover can increase infiltration and reduce run‐off in drylands (Cerdà & Rodrigo‐Comino, 2020). Riparian corridors may improve water quality through the stabilization of stream banks and reduction of run‐off to waterways (Abernethy & Rutherford, 1999; Hansen et al., 2010). However, pasture reforestation can sometimes decrease soil carbon (Guo et al., 2008), and in some cases, silvopastoral systems can have lower SOC than open pastures (Douglas et al., 2020). The interaction between tree species, soil type and climate has a significant influence on SOC dynamics, and should be considered in GHG accounting for agroforestry (Douglas et al., 2020).

Trees can also enhance the biodiversity of grazing systems (Table 2), particularly where native species are planted. Scattered trees in paddocks or fields and living fences play a key role in ecosystem function and biodiversity conservation in highly modified landscapes (Manning et al., 2006). Plantings that include understorey trees and shrubs increase structural and species diversity, improving habitat value for fauna including pollinators and biological control agents. When strategically located relative to remnant vegetation, reforestation with native species can enhance landscape connectivity, creating biological corridors that may support adaptation, facilitating movement of species in a changing climate (Beier et al., 2012). Trees on farms can improve biodiversity and ecosystem functions indirectly by reducing pressure on natural forests from fuel wood extraction (Table 2).

While there are synergies between reforestation and livestock production, there are trade‐offs with agricultural production as tree basal area increases (Table 2; Cerdà & Rodrigo‐Comino, 2020). There are also trade‐offs between environmental co‐benefits: CS is often higher in tree monocultures, while biodiversity value and resilience are greater in mixed species plantings that include shrubs (Paul et al., 2016).

## 8 THE NEED FOR INTEGRATED, CROSS‐SECTOR, MULTI‐METRIC, MULTI‐SCALE AND HOLISTIC SYSTEMS‐BASED EMISSIONS MITIGATION FOCI

While assessment of the *technical* potential for mitigation is a useful first step towards quantifying the *feasible* mitigation associated with an intervention, future studies must go broader to examine the co‐benefits (synergies) and trade‐offs (antagonisms, both unavoidable and unexpected) that exist within and between:

1. **Sectors:** for example, monogastric versus ruminant livestock (Sections 2, 3 and 4);
2. **Disciplines:** for example, social, economic, environmental, biophysical, political, institutional etc. (Section 5);
3. **Metrics:** for example, GHG emissions per unit product, per unit protein, per unit animal etc. (Sections 8 And 9);
4. **Industry and value chains**: for example, primary producers, retailers, wholesalers, processors and consumers (Section 8);
5. **Scales:** for example, farm, region, country, continent, global (Section 10); and
6. **Processes and systems**, considering holistic systems, rather than reductionist approaches that focus on one element while holding all others constant (Section 8).

We are not advocating that future studies should be exhaustive, but rather that *future work give consideration to multidimensional aspects*. This could include computing alternative metrics of GHG emissions intensity; assessing the cost, environmental and social aspects of a given intervention; discussing the physical, financial and regional implications of adopting GHG emissions interventions; examining emissions leakage across industries; or quantifying trade‐offs between the goals of farmers (often profitability rather than productivity) versus those of governments (to ensure productivity and thus food security).

Poore and Nemecek (2018) note that monitoring a single variable or proxy is often a poor predictor for net GHG emissions. They suggested that concurrent assessment of multiple impacts and avoiding proxies supported far better decisions, helping prevent harmful, unintended consequences. For example, adding N fertilizer to increase pasture biomass may result in pulses of N_2_O that when cumulated can negate SOC benefit (Garnett et al., 2017). The environmental and social importance of different impacts varies locally, given land scarcity, endemic biodiversity and water quality, among other factors (Poore & Nemecek, 2018), suggesting multiple proxies are necessary to quantify key sustainability indicators in the social, environmental and economic dimensions.

We showed in Figure 6 that assessments of social aspects of GHG emissions mitigation are particularly lacking. Salmon et al. (2018) suggested that the consideration of the diverse objectives of stakeholders and agricultural paradigms should inform the assessments of how the livestock sector in LMICs will evolve with future (and growing) demand for livestock products. Salmon et al. (2018) identified intensification trade‐offs related to economic gains, gender equity, environmental concerns, human nutrition and food safety. They underscored the need to consider the distribution of benefits between *individuals* versus the average benefit is derived for a *population*.

Holistic, systems‐based analyses are useful for examining feedback loops, leverage points, cross‐scale antagonisms or synergies and emergent properties (Alcock et al., 2015; Chang‐Fung‐Martel et al., 2017; Harrison et al., 2011a). Systems‐based assessments can be used in a whole‐farm sense (Ahmed et al., 2020; Phelan et al., 2015; Rawnsley et al., 2018) and across the value chain to design interventions for reducing emissions associated with food loss and waste (Galford et al., 2020). Life cycle assessments are useful in analysing and attributing the emissions of a product from the ‘cradle to grave’ (or from resource extraction to waste disposal, see Section 10). Because system assessments enable an analysis of interconnected processes, they are useful for determining leverage points at which a small change within a system could lead to larger changes in behaviours. Leverage points are thus one of the most critical points of the system for intervention (Weinberger et al., 2015).

**FIGURE 8:**
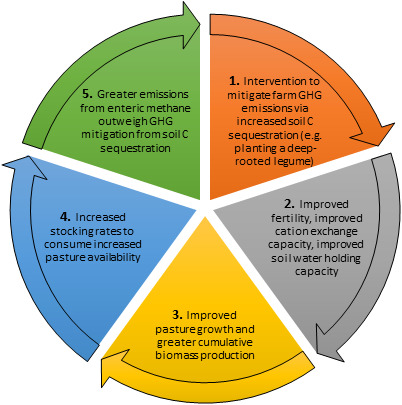
Example of a trade‐off resulting from an intervention aimed at increasing soil carbon (e.g. through participation in an emissions trading scheme) that could result in higher net GHG emissions compared with business as usual. In the absence of external influence or constraints, landholders are likely to adapt grazing management to utilize any additional pasture or grassland production. This example illustrates the importance of assessing GHG emissions mitigation options holistically to manage unforeseen trade‐offs, such as pollution swapping

System‐based assessments can help bridge knowledge gaps across scales, from farm (Christie et al., 2018; Harrison, Christie, et al., 2014; Harrison, Jackson, et al., 2014; Pembleton et al., 2016) to regional and continental levels (Chang et al., 2021). At the farm scale, soil carbon is often proposed as an avenue for GHG emissions mitigation (Section 6). However, holistic assessment of other concurrent changes within a farm system may find that interventions to improve soil carbon can also increase whole farm emissions due to unexpected feedbacks wherein improvements in soil carbon raise pasture productivity (e.g. Rawnsley et al., 2018), leading to greater stocking rates and thus increase net GHG emissions at the farm scale (Figure 8). Nevertheless, increased production intensity can provide opportunities for land sparing at farm or regional levels through the displacement of less efficient production elsewhere, in which case they would be consistent with global climate change mitigation goals.

Holistic, system‐based assessments provide a pathway to *transcend multiple scales*. Chang et al. (2021) estimated regional patterns and global trends using a spatially explicit land surface model and showed that human activities (rather than indirect effects of climate change) were responsible for causing managed grasslands to switch from a source to a sink of GHG emissions. The transition was mainly due to increased livestock numbers and accelerated conversion of natural lands to pasture. However, climate change also contributed to increased SOC sequestration as a result of increased productivity of grasslands due to increased atmospheric CO_2_ and nitrogen deposition. Chang et al. (2021) showed that the net radiative forcing of global grasslands has been increasing since the 1960s but is currently close to neutral, because net global climate warming caused by managed grassland and ruminant livestock cancels the net climate cooling from carbon sinks. System‐based approaches such as that used by Chang et al. (2021) provide an avenue for assessing the combined potential of multiple mitigation options imposed simultaneously (Box 2), facilitating insights into expected and unforeseen co‐benefits and trade‐offs.

BOX 2 **Combining or ‘stacking’ multiple GHG mitigation interventions**

Although GHG mitigation options have received much attention in isolation, less work has been conducted on intervention ‘stacking’ (i.e. practices in which two or more GHG mitigation options are applied simultaneously). When applied holistically, concurrent assessment of multiple metrics can be used to evaluate the overall promise of ‘bundled’ or ‘stacked’ GHG mitigation options. For example, Harrison, Cullen, Tomkins, et al. (2016) examined the nexus between GHG emissions, production and profitability for extensive beef farming systems in central Australia. They found that stacking of GHG mitigation options by mating maiden heifers earlier, optimising herd structure and increasing the fecundity of breeding cows resulted in higher gross margins than any of the three interventions applied individually. Adding the perennial legume leucaena (*Leucaena leucephala*) reduced gross margins but resulted in the lowest emissions intensity due to the positive effects of leucaena on liveweight gain and emissions mitigation (lower enteric methane and greater soil organic carbon sequestration). Similarly, Beukes et al. (2010) suggest that profitability of New Zealand dairy farms would be improved through the implementation of crossbred cows with higher genetic merit and reproductive performance, higher pasture quality, introduction of supplementary feeds (maize silage) and the application of a nitrification inhibitor under wet conditions to reduce N_2_O emissions. Beauchemin et al. (2020) similarly indicate that a combination of mitigation strategies would be necessary to achieve substantial mitigation of enteric methane.

While the studies above examined the profitability of combined interventions, the costs of transitioning to a new system (e.g. to cows with greater genetic merit and/or reproductive performance) were not considered. Instead, the studies above examined the final steady‐state scenarios after interventions were imposed. Given that significant and multi‐faceted farming systems transitions can take time to be fully realised, such economic and biophysical considerations should be accounted for in future studies of this type.

Simultaneous improvements in production system (or business model) can be thought of as another example of intervention stacking. Steinfeld and Gerber (2010) concluded that in many LMIC, changing production systems from ruminants to monogastrics and continued efficiency gains in the production of feed and livestock could significantly attenuate the environmental impacts of livestock production systems. They indicated that although addressing excessive levels of consumption will help reduce environmental impact, there is a vast mitigation potential on the production side. Addressing environmental impacts of livestock on the production side may also carry important benefits for socially and economically disadvantaged livestock producers in LMIC.

Herrero et al. (2021) indicate that ‘sociotechnical innovation bundles’ (i.e. appropriately contextualised combinations of science and technology advancements coupled with institutional or policy adaptations that show promise for GHG mitigation) combined with institutional or policy reforms guided by a consistent mission or intentionality (Klerkx & Begemann, 2020) may be able to address multidisciplinary challenges and mitigate unintended consequences. Only with such concurrent policy reforms and other appropriate changes to the system will potential GHG mitigation be realised.

## 9 ARE SUSTAINABILITY METRICS FOR LIVESTOCK PRODUCTS APPROPRIATE, COMPARABLE AND SUITABLY CROSS‐DISCIPLINARY?

Future research must shift from the prevailing and parochial view on carbon (‘carbon myopia’) towards more harmonized and globally reconciled environmental, economic and social assessments. The derivation of sustainability methods and metrics in this way requires iterative input from multiple stakeholders, including farmers, industry and the scientific community, to civil society and governments at all levels. The application of holistic sustainability assessments would minimize risk that positive progress in one area (such as emissions mitigation) results in maladaptation in other dimensions, regions or sectors (such as diminished food security or natural habitat destruction).

As a first step towards more holistic sustainability comparisons, future work should quantify multiple metrics simultaneously: both within disciplines (alternative emissions metrics) and across disciplines, combining biophysical, social and economic indicators to examine livestock production from a social–ecological system perspective. The evolving science of global warming potential metrics is a case in point (Box 3): Choice of metric for assessing climate impacts of CH_4_ affects the level of responsibility for emissions reduction between countries, sectors and communities. This decision requires consideration of fairness, incentives and economic feedbacks, in addition to direct climate effects.

BOX 3 **The state of the art in global warming metrics: Are we comparing apples with apples?**

Predominant GHGs in livestock systems include CO_2_, CH_4_ and N_2_O. Methane is a powerful GHG but has a short atmospheric lifetime (~12 years) compared with 120 years for N_2_O, and well over 500 years for CO_2_ (Myhre et al., 2013). Following convention applied in national inventory reporting to the UNFCCC, global warming potential (GWP) has become an accepted metric for comparison of GHG emissions across agricultural products. GWP is calculated as the radiative forcing of a given GHG integrated over a chosen period (usually 100 years) expressed relative to the effect of an emission of CO_2_ with units of CO_2_‐equivalents (CO_2_‐e).

While cumulative CO_2_ emissions dominate anthropogenic warming over centuries, temperatures over the coming decades are also strongly influenced by short‐lived climate pollutants (SLCP) including methane (Allen et al., 2018). Alternative GHG emission metrics have thus been proposed, including global temperature change (GTP) potential (Allen et al., 2016; Shine, 2009; Shine et al., 2007) and GWP* (Allen et al., 2018), which place alternative weighting on short‐lived GHGs compared with GWP. GTP compares GHGs based on their effect on global mean surface temperate at the end of the chosen time horizon. GWP* compares CO_2_ emissions to date with the current rate of emissions of SLCP, so to be computed requires two emissions measurements over a period of time (Δt)—usually 20 years in the case of CH_4_. Proponents argue that GWP* better reflects the shorter atmospheric lifetime but relatively higher radiative forcing of gases like CH_4_, accounting the effects of both long‐lived and short‐lived pollutants on radiative forcing and temperatures over a wider range of time scales (Allen et al., 2018).

But GWP* also has limitations. The metric may penalise growth in livestock numbers, such as that anticipated in LMIC where livestock are critical for food security, and would give a negative CO_2_‐equivalent value in contexts where there is a static or declining livestock populations (e.g. Australia); the latter implying cooling, which is clearly not the case. The relevant application of GWP* to inform mitigation policy is thus contested, with concerns raised over equity implications (Harrison, Jackson, et al., 2014; Rogelj & Schleussner, 2019).

Debate surrounding GHG equivalence reinforces the need for further development of metrics that facilitate more equitable approaches for comparing agricultural products—in particular, comparing short‐ and long‐lived GHGs—but also in the context of offsets, for quantifying equivalence of carbon sequestration against GHGs with various lifetimes and differing impacts on the atmosphere. This is critically important, given the implications using GWP* may have in considering ‘carbon neutral’ or ‘net zero emissions’ targets for sectors emitting different GHG compositions (Lynch et al., 2020).

The development of such metrics would be expected to lead towards the derivation of functional units that could be applied to measure GHG emissions and account for pollution swapping, avoiding ‘trivial solutions to a global problem’ (Franks & Hadingham, 2012).

While there has been much work on the issue of GHG emissions allocation between products from the same animal (e.g. the partitioning of emissions between wool and meat for sheep, or between meat and milk for dairy cattle (e.g. Eady et al., 2012; Rice et al., 2017; Wiedemann et al., 2015)), there is less work on the appropriateness of emissions metrics for comparison of the relative GHG emissions intensities of livestock systems. This includes both the metric per se and the number of metrics used to make any one comparison. By way of example, Cottle et al. (2016) modelled sheep GHG emissions intensities in 28 locations across Australia and showed that per animal (dry sheep equivalent), GHG emissions intensities were lower in cool temperate regions near the coast. In contrast, when emissions were computed per hectare, inland arid and semi‐arid regions had lower emissions intensities. Such contradictions highlight a need to examine multiple metrics, including both emissions intensities and net emissions on a per land area and per animal basis. Emissions intensities are important when comparing production systems or enterprises (as in Cottle et al., 2016), and net emissions are important when calculating the relative effect of a production system on the atmosphere (and thus climate change mitigation potential).

Sustainability assessments across disciplines are also key to the development of more appropriate comparisons of the sustainability of agricultural systems going forwards. Evaluation of alternative emissions reduction strategies for livestock systems, and comparison with alternative livelihood systems, land uses and food production systems, should take an integrated systems approach (Bierbaum et al., 2018; O'Connell et al., 2019) that considers not just emissions but also environmental indicators, such as effects on soil acidification, water eutrophication, fresh water withdrawals and other ecosystem functions, as appropriate to context. In a globally reconciled and methodologically harmonized study, Poore and Nemecek (2018) showed that while GHG emissions intensities of lamb and mutton were higher than those of farmed crustaceans (10–25 kg cf. 5–15 CO_2_‐e/100 g protein), effects of lamb/mutton production on acidification and eutrophication were much smaller than that of farmed crustaceans (50–75 cf. 50–150 g SO_2_‐e and 10–55 cf. 50–150 g PO_4_^3—^e respectively). Similar parallels could be drawn with scarcity‐weighted water withdrawals for beef, which in many cases were much lower than those of nuts and grains.

These examples suggest that if acidification and/or eutrophication were attributed a higher priority than GHG emissions in future assessments of sustainability, proponents for food production systems, policy and/or potentially societal preferences may shift favour towards red meat and away from farmed crustaceans. These policy shifts may then be at the expense of air quality (Domingo et al., 2021) and global warming if the net emissions from livestock were greater than those associated with crustaceans. Comparisons with other alternative protein sources, such as plant‐based synthetic meat, microbial protein and cultured meat, have similarly identified trade‐offs between ER and inputs of energy, land, water and nutrients (Hu et al., 2020; Lynch & Pierrehumbert, 2019; Smetana et al., 2015; Spiller et al., 2020). *Consequential life cycle assessments* (CLCA) potentially represent a more integrated and arguably superior protocol for comparing sustainability dimensions of alternative protein sources as opposed to approaches that focus on carbon emissions alone.

‘Wicked problems’ of this type elicit questions on the priority placed on sustainability indicators, as a focus on one metric at one point in time (e.g. ‘carbon myopia’) could be at the detriment of another sustainability dimension in future (e.g. water quality, nutrient leaching, soil erosion, loss of biodiversity etc.). Similarly, changing priorities over time may shift societal and political preferences from one area of perceived importance to another, potentially negating years of previous effort and achievement in other sustainability dimensions. The priority placed on any sustainability indicator will be largely influenced by stakeholder interests. It is critically important that a representative range of stakeholders engage in constructive, iterative dialogue that results in the identification of new sustainability priorities, because perceived ‘sustainability’ will very much depend on the interests, background, education and potential benefit of those who contribute to the formation of any new policy. This type of risk could be envisioned with a change of government that shifts their prevailing focus from carbon emissions mitigation to sustainable intensification; while enhanced food production and security may ensue, other societal outcomes may be maladaptive (e.g. causing environmental degradation, financial instability, insecurity of land tenure or adverse human health outcomes) if they are not given appropriate and concurrent consideration.

We propose that future evaluation of emissions mitigation strategies for livestock systems includes comparison with alternative livelihoods, land uses and food production systems, applying an integrated systems approach (Bierbaum et al., 2018; O'Connell et al., 2019). Design of sustainable development interventions and governance systems should simultaneously consider *multiple objectives and metrics* to minimize trade‐offs, stimulate co‐benefits, enhance efficiency in policy implementation and promulgate more sustainable outcomes (Cowie et al., 2007; Tengberg & Valencia, 2018). In line with our results in Figure 6, these sentiments suggest that greater understanding of social and economic contexts is critical to better inform landholder engagement and extension programmes, and more effectively design policy incentives to enhance adoption of mitigation strategies.

## 10 IN PURSUIT OF MULTI‐SCALE TRANSDISCIPLINARY SOLUTIONS: CONSEQUENTIAL LIFE CYCLE ASSESSMENTS AND ‘SOCIO‐ECONOMIC PLANETARY BOUNDARIES’

As discussed above, system‐based and holistic assessments may provide a prospective way forward for capturing multiple feedbacks within connected systems, including synergies, antagonisms and emergent properties. A system‐based approach frequently used to quantify carbon footprints associated with food production that accounts for some environmental trade‐offs and co‐benefits is *life cycle assessment* (LCA; e.g. Alvarez‐Hess et al., 2019; Beauchemin et al., 2010; Browne et al., 2011; Casey, 2005; Reijnders, 2012). Many ‘conventional’ or ‘attributional’ LCAs have shown that ruminant production has a GHG emissions intensity higher than that of crop production (Browne et al., 2011; Clune et al., 2017; Tuomisto & Teixeira de Mattos, 2011) and that livestock require several times the amount of land compared with the production of vegetable proteins (Smith et al., 2013; Stehfest et al., 2009).

While product‐focussed attributional LCAs show the relative impact of functionally equivalent food products, they overlook the *raison d'être* of multifunctional land use systems, as well as the consequences of policy and consumer choice. For example, attributional LCA of dairy milk shows that subsistence dairy production in LMIC has higher emissions intensity than milk produced in a highly intensive dairy production system (e.g. compare the emissions intensity of African and European milk in Figure 4). However, as explained above, livestock in smallholder systems serve many functions, including milk and meat production and draught power, but also provide less tangible social values, such as use for dowry, demonstration of prestige or wealth and as key pillars of cultural identity (see Table 2 and Weiler et al., 2014). When GHG emissions were allocated between these various purposes, Weiler et al. (2014) found GHG emissions per unit weight of milk from smallholder dairying in Kenya of 2.0 (0.9–4.3) kg CO_2_‐e using economic allocation to food products only, 1.6 (0.8–2.9) kg CO_2_‐e when allocation was based on economic functions, and 1.1 (0.5–1.7) kg CO_2_‐e when emissions allocation considered the livelihood values of livestock. These emissions intensities are comparable to intensive dairy production systems (Alvarez‐Hess et al., 2019; Beauchemin et al., 2010; Christie et al., 2018; Galloway et al., 2018), clearly highlighting a need for careful consideration of the diverse economic, environmental and socio‐economic roles that livestock play, particularly in smallholder systems.

Consequential life cycle assessment (CLCA) is emerging as a tool for capturing environmental impacts of production systems that go beyond physical relationships accounted for in conventional or attributional LCA (Earles & Halog, 2011). CLCA aims to describe how physical flows can change as a consequence of an increase or decrease in demand for the product under study. This is an important advancement on attributional LCA methods, particularly when applied to GHG emissions from the livestock sector in developing countries.

Consequential LCA has been used to assess emissions associated with consumer preferences in developed countries. Recent assessments have considered emissions associated with land use change resulting from dietary shifts in red meat consumption towards synthetic meat: There may be more pastoral and crop land available for reforestation in moving systems away from red meat, but conversely, an increase in crop production to supply the nutrients for plant‐based meat substitutes could lead to long‐term grasslands being cropped, losing substantial SOC and increasing N_2_O emissions (Palmer et al., 2017; Pinto et al., 2004). A subsequent study showed that while cultured meat initially resulted in less climate warming than that from a cattle production system, the emissions gap narrowed in the long term (Lynch & Pierrehumbert, 2019). The same study revealed that in some cases, cattle systems resulted in significantly less warming than cultured meat because CH_4_ emissions from ruminant production were short‐lived, in contrast to the long‐lived and fossil fuel‐derived CO_2_. This insight again gives credence to the need for continuing investigation of alternative global warming metrics described in Box 3. A shift from livestock to cropping in LMIC may require more diesel use for mechanized cultivation as well as conventional fertilizers, increasing CO_2_ and N_2_O emissions. A transition away from mixed farming systems—particularly in subsistence agriculture—could also compromise local food security and resilience to climate change. While conventional LCA has been useful for exploring the comparative footprint of agricultural products, future policy would be better served by a more comprehensive and inclusive CLCA approach to better understand possible outcomes of policy choices (Brandão et al., 2017; Plevin et al., 2014).

**FIGURE 9:**
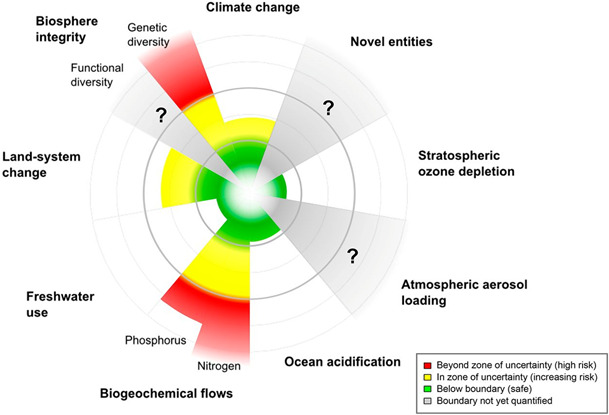
Current status of the nine planetary boundaries. Below the green zone defines the safe operating space, yellow represents the uncertainty zone (increasing risk), red is the high‐risk zone. The planetary boundary lies at the innermost heavy circle. The control variable for climate change is equivalent atmospheric CO_2_ concentration. Processes for which global‐level boundaries are not quantified are shaded grey (atmospheric aerosol loading, novel entities and the functional role of biosphere integrity). Reproduced with permission from Steffen et al. (2015)

A multiscale approach broader than CLCA would be an expansion of the ‘planetary boundaries’ framework (Häyhä et al., 2016). The ‘planetary boundaries’ paradigm was developed assuming that transgression of one or more planetary boundaries could be deleterious and potentially catastrophic to humanity due to risks associated with crossing thresholds triggering non‐linear, abrupt environmental tipping points within continental‐scale to planetary‐scale systems (Rockström et al., 2009). The framework defines a ‘safe operating space for humanity’, moving away from sectoral analyses of limits to growth aimed at minimizing negative externalities, towards estimation of a safe space for human development. Planetary boundaries define, as it were, the boundaries of a “planetary playing field” for humanity if major global human‐induced environmental change is to be avoided (Rockström et al., 2009). Two of the planetary boundaries have already been transgressed (biosphere integrity and biogeochemical flows; Figure 9), with some authors suggesting that agriculture has been a major driver of this transgression (Campbell et al., 2017).

Planetary boundaries were originally conceived for nine key areas of the earth system (Figure 9), but the work received criticism in failing to account for trade‐offs between planetary boundaries (Lade et al., 2020). Expansion of the planetary boundaries to encompass socio‐economic, cultural and ethical factors (Häyhä et al., 2016) would seem pertinent, as would approaches for accounting for interactions between planetary boundaries (Lade et al., 2020).

A greater focus on the development of food systems within a *‘socio‐economic planetary boundaries’ systems‐based paradigm* would be a fruitful endeavour for future comparisons of the sustainability of livestock production systems. Such focus may help shift the dialogue from a predominant and myopic focus on the technology aspects of GHG emissions mitigation to a multi‐metric, multi‐scale, transdisciplinary scenario analysis process. The development of such scenarios would help develop a positive visioning for the future, ensuring harmony across disciplines and global consistency in policy development.

The development of socio‐economic planetary boundaries in this way should support and foster stakeholder dialogues, learning and understanding by visualizing possible futures. Such scenarios would provide narratives to describe how a practice in a particular region may look (Weinberger et al., 2015) and could be used to develop a positive vision for future sustainable development.

## 11 THE NEED FOR CONSISTENT, SUSTAINED AND LONG‐TERM RESEARCH AND DEVELOPMENT FUNDING COMMITMENTS FOR REDUCING LIVESTOCK GHG EMISSIONS

Methane and nitrous oxide production are microbially mediated processes that have evolved over 50 M years. The development of mitigation strategies that fundamentally change these processes is thus a significant challenge, requiring sustained research commitment and funding continuity for research groups addressing these issues. Investment for R&D to reduce agricultural emissions intensities has a high benefit:cost ratio, ultimately improving economic efficiencies, reducing both poverty and agricultural emissions (Laborde et al., 2021). Despite this, many countries operate with short‐term, intermittent research and development (R&D) funding. In Australia, for example, R&D investment in agricultural GHG emissions mitigation and climate change has historically been highly variable, resulting in recruitment then jettison of academic and institutional research capacity with each funding cycle. Agricultural R&D funding cycles in LMIC tend to be even more variable, often due to the short‐term project‐oriented nature of donor and development bank funding (Beintema et al., 2012). Such lack of continuing R&D investment has contributed to the paucity of GHG emissions mitigation technologies available for LMIC, as noted above. Considering the projected growth in livestock production and GHG emissions in LMIC in the next few decades, the absence of steady, sustained R&D funding heralds a dire outlook for enduring GHG emissions mitigation in developing nations.

## 12 CONCLUDING REMARKS

The purpose of this review was to (1) examine the global distribution of livestock production and GHG emissions, (2) explore the co‐benefits and trade‐offs associated with economic, environmental and social implications of GHG emissions mitigation, (3) critique current approaches used to quantify and report livestock GHG emissions and (4) highlight opportunities for future GHG emissions mitigation research.

This review has helped distil many insights and opportunities for pathways forward:

1. Globally, livestock production systems exist for several reasons, many of which are critical to livelihoods. In many areas, livestock are needed to satisfy a variety of human needs. In addition to production of meat, milk, eggs, wool, hides and skin, livestock provide draught power and nutrient cycling, supporting the environmental sustainability of production (Steinfeld et al., 2003). Ruminant livestock utilize non‐arable land, converting fibrous and cellulosic materials into edible human protein. In many LMIC, livestock constitute the main (if not only) household capital reserve, serving as a strategic financial reserve that reduces risk and adds financial stability to the farming system (Steinfeld et al., 2003).
2. GHG emissions associated with ruminant livestock are greatest in LMIC. In contrast, the majority of GHG emissions mitigation research has been undertaken in—and designed for—developed countries. Currently, the developing world contributes 66% of global GHG emissions from ruminants: between 2017 and 2029, 80% of the growth in global meat production will occur in LMIC. The limited GHG emissions mitigation research and highly volatile R&D funding for such work in LMIC limits research capability and progress towards GHG emissions mitigation goals. Taken together, these trends underscore a dire need for research, development, extension and adoption of emissions mitigation technologies, skills and practices in LMIC. This is particularly so given the critical biophysical, cultural, financial and social dependencies of many smallholders on livestock.
3. The majority of GHG emissions mitigation research predominantly (in some cases myopically) focuses on technology aspects of GHG emissions mitigation. By corollary, there is a paucity of studies that concurrently assess social, economic and environmental aspects of GHG emissions mitigation. Of the 54 livestock GHG emissions mitigation interventions examined, only 16 had triple bottom‐line benefits and medium–high mitigation potential. These included manure management, higher fecundity/weaning rates, increasing animal growth rates and reducing time to slaughter. Planting deep‐rooted legumes offers multiple avenues for emissions mitigation through enteric CH_4_ mitigation as well as soil CS. The international scientific community must work to understand the wider social, economic and environmental co‐benefits and trade‐offs caused by the implementation of such interventions, because any change to existing practices could result in maladaptive outcomes, such as compromised food security or financial stability.
4. While multi‐pronged efforts aimed at both emissions reduction (ER) and carbon sequestration (CS) are likely to achieve greater emissions mitigation, CS opportunities may provide greater potential for social, economic and environmental co‐benefits. Strategic, thoughtful planting of trees and shelter belts in areas of low pasture productivity paves avenues for CS but also heralds significant co‐benefits, such as the provision of shade and shelter for livestock, habitat for native flora and fauna, fuel and firewood in LMIC and building of natural capital.
5. In addition to large areas of agroforestry that have already been adopted in LMIC, there appears to be considerable potential for further CS in soils and vegetation in LMIC. Despite the array of co‐benefits that come with such emissions mitigation strategies, implementation of soil or vegetation measures specifically for GHG emissions mitigation through participation in carbon markets in LMIC has historically been hindered due to relatively high transaction costs and social barriers such as insecurity of land tenure. Improved adoption of practices for improving carbon stocks in soils and vegetation will require the implementation of both appropriate policy legislation and accompanying socio‐economic developments (Kongsager et al., 2013).
6. The scientific community needs to shift from the frequent reductionist, unicentric focus on carbon, to more holistic, systems‐based, multi‐metric and transdisciplinary approaches. Such shifts will help uncover feedback loops, leverage points, cross‐scale antagonisms/synergies and unforeseen emergent properties. Mainstream attention needs to progress from the CO_2_‐e mitigation lens in isolation to the investigation of multiple sustainability indicators that lead to profitable, practical and socially acceptable solutions with cross‐sectoral and multi‐scale advantages. The derivation of harmonized, globally reconciled sustainability metrics will require iterative dialogue from stakeholders at all levels, from farmers, industry and the scientific community to civil society and governments. The development of sustainability metrics in this way would be expected to limit cases in which positive progress in one area results in transgressive outcomes in other areas.
7. ‘Wicked problems’ in food sustainability elicit questions on how priority is placed on sustainability indicators, as a focus on one metric (e.g. carbon or GHG emissions) could be to the detriment of other sustainability metrics (e.g. water quality, nutrient leaching, soil erosion, loss of biodiversity etc.). Evolving priorities over time may shift societal and political preferences from one area of perceived importance to another. Shifts in industry priorities, governance and policy in this way could negate years of previous work in other areas. Risks of this type could be envisioned with a change to governments that are less interested in emissions mitigation and more focussed on sustainable intensification.
8. Nascent approaches such as consequential life cycle assessments (LCA) and socio‐economic planetary boundaries provide opportunities to advance the state of the art of GHG emissions mitigation. In contrast to traditional attributional LCA, consequential LCA captures the wider environmental and socio‐economic impacts of production systems, such as nutritional value, draught power, social status and financial capital provided by livestock systems in smallholder systems.
9. Integration of a system‐based paradigm within a ‘socio‐economic planetary boundaries’ framework provides another prospective pathway forward. This approach may yield fruitful comparisons of the sustainability of various livestock production systems and may help shift the dialogue from an individual technology and carbon emissions mitigation lens in isolation (our phrase *carbon myopia*) towards a more nuanced global discussion, facilitating decision‐making through refinement of alternative scenarios, facilitating harmony across disciplines and consistency in global objectives.

## Data Availability

Any data produced by the authors in this article will be made available online upon request.

## Footnotes

^1^
Noting caveats associated with global warming potentials explored later in this article
